# Editorial: Molecular, Cellular and Model Organism Approaches for Understanding the Basis of Neurological Disease

**DOI:** 10.3389/fnmol.2017.00074

**Published:** 2017-03-20

**Authors:** Robert J. Harvey, Kirsten Harvey

**Affiliations:** Department of Pharmacology, UCL School of PharmacyLondon, UK

**Keywords:** next-gen sequencing, genetics, cell line, zebrafish, mouse

Next-generation sequencing technologies have resulted in remarkable increases in our understanding of human neurological disorders through the identification of disease-causing or protective sequence variants. However, in many cases, new molecular, cellular, and whole-organism models are required to understand how changes at the DNA, RNA, or protein level affect neuronal and synaptic function. In turn, these models may enable understanding of key disease processes and the identification of new targets for the medicines of the future. What is also evident is the high quality and impact of the research conducted in this field. This is reflected in the reviews and research articles in this Special Issue entitled “*Molecular, cellular and model organism approaches for understanding the basis of neurological disease*.”

Mutation and gene discovery in neurological diseases has recently been transformed by large-scale DNA sequencing approaches coupled with stringent variant filtering. This is particularly true of intellectual disability, characterized by significantly impaired intellectual and adaptive function. Long et al. and Kalscheuer et al. highlight the utility of this methodology by resolving two families with X-linked intellectual disability caused by mutations in *ARHGEF9* and *IQSEC2*, which both encode neuronal GDP–GTP exchange factors. One important aspect of these studies was functional validation of the pathogenic variants using molecular modeling, phosphoinositide binding, gephyrin clustering, or GDP–GTP exchange activity. By contrast, Krüger et al. reported on the use of panel sequencing to study a cohort of 80 German patients with Amyotrophic lateral sclerosis (ALS), a rapidly progressive, fatal neurological disease that causes degeneration of motor neurones. This approach allowed deep sequencing of 39 confirmed ALS genes and candidate genes, as well as 238 genes associated with other neurodegenerative diseases. They identified 79 rare potentially pathogenic variants in 27 ALS-associated genes, as well as pathogenic hexanucleotide repeats in *C9orf72*. Based on this study, the authors recommend two-staged genetic testing for ALS in patients with familial and sporadic ALS, comprising *C9orf72* repeat analysis followed by comprehensive panel sequencing.

It is also important to remember that not all rare genetic variants are disease causing. Nixon-Abell et al. reported the detailed molecular and cellular characterization of a protective variant in *LRRK2*, encoding leucine-rich repeat kinase 2 (LRRK2) which is intimately associated with the pathogenesis of Parkinson's disease. Importantly, the p.R1398H variant affects GTPase function, axon outgrowth, and Wnt signaling in a manner opposite to pathogenic *LRRK2* mutations. The authors concluded that LRRK2-mediated Wnt signaling and GTPase function are fundamental in conferring disease susceptibility, and that this has clear implications for future therapeutic interventions in Parkinson's disease.

Sophisticated cellular models of disease are increasingly vital as high-throughput screening tools for recreating events at normal and disrupted synapses *in vitro*. Kuenzel et al. reported a new tool for screening of *in vitro* neurotoxicity (NT) and developmental neurotoxicity (DNT) mediated by inhibitory γ-aminobutyric acid type A (GABA_A_) and glycine receptors (GlyRs). They generated a human pluripotent stem cell line (NT2) that stably expresses YFP^I152L^, a halide-sensitive variant of YFP, which allows for fluorescence-based functional analysis of Cl^−^ channels. Importantly, upon stimulation with retinoic acid, these NT2 cells undergo neuronal differentiation, allowing pharmacological and toxicological evaluation of native GABA_A_Rs and GlyRs at different developmental stages. By contrast, Dixon et al. reported an “artificial synapse” system—a neuron-HEK293 cell co-culture technique for generating inhibitory synapses incorporating defined combinations of wild-type or mutant GABA_A_R or GlyR subunits. As well as allowing control over the subunit composition of the GlyRs under study, the electrotonically compact shape of HEK293 cells combined with rapid agonist application allows IPSC waveforms to be resolved with high fidelity using electrophysiology.

The role of the GlyR M3-M4 intracellular domain in health and disease was also reviewed by Langlhofer and Villmann who comprehensively documented molecular determinants of phosphorylation, intracellular sorting, protein–protein interactions, subunit topology, and modulation by G proteins, ethanol, and cannabinoids. Barral and Kurian also reviewed the current and future potential of patient-derived induced pluripotent stem cells (iPSCs) in the field of childhood neurological disorders. Importantly, iPSCs can now be routinely differentiated into specific neuronal subtypes which represent valuable *in vitro* models of disease. They are also vital tools for testing existing drugs with repurposing potential, or novel compounds and gene therapies, which then can be translated to clinical practice. Imaging also has a key role to play in diagnosis of disease. Poutiainen et al. reviewed advances in non-invasive imaging techniques such as positron emission tomography (PET) and prospective biomarkers for the diagnosis of multiple sclerosis.

Whole-organism *in vivo* models also continue to make an impact in this field. Kozol et al. and Ogino and Hirata reviewed the many advantages of zebrafish in the study of neurological disease. Validation of new disease genes and mutations is now possible through CRISPR/Cas9 mutagenesis of zebrafish gene orthologs, which rapidly creates new disease models which are amenable to phenotyping and high-throughput drug screening. Watanabe et al. also highlighted the use of sophisticated mouse models, showing how doxycycline-sensitive, Cre-mediated gene ablation of NMDA receptors in hippocampal excitatory neurons results in neurodegeneration in aging mice. This study highlighted the potentially damaging effects of long-term administration of NMDAR antagonists for therapeutic purposes. Lykhmus et al. used an acute model of LPS-induced neuroinflammation in mice and an *in vitro* model in cultured glioblastoma U373 cells to study neuroinflammation, which accompanies and often precedes the development of neurodegenerative pathologies such as Parkinson's and Alzheimer's diseases. They found that acute LPS-induced inflammation induced the cholinergic anti-inflammatory pathway in the brain, with down-regulation of α7 subunit-containing nAChRs limiting these effects. Curiously, nAChR α7 subunit-specific antibodies aggravated neuroinflammation, by inducing the pro-inflammatory interleukin-6 and dampening anti-inflammatory miRNAs. However, the authors also highlighted that nAChR α7-specific antibodies may protect brain mitochondria and decrease the levels of pro-apoptotic miRNAs, preventing LPS-induced neurodegeneration.

We thank all contributors for their interesting and informative articles and the reviewers for their constructive and thoughtful suggestions.

## Author contributions

KH and RJH wrote the manuscript and both authors approved the final version for publication.

### Conflict of interest statement

The authors declare that the research was conducted in the absence of any commercial or financial relationships that could be construed as a potential conflict of interest.

